# Informed consent and ethics committee approval in laboratory medicine

**DOI:** 10.11613/BM.2018.030201

**Published:** 2018-10-15

**Authors:** Ana Borovecki, Ana Mlinaric, Martina Horvat, Vesna Supak Smolcic

**Affiliations:** 1 “Andrija Štampar” School of Public Health, University of Zagreb School of Medicine, Zagreb, Croatia; 2 Research Integrity Editor, *Biochemia Medica*; 3 Department of Laboratory Diagnostics, University Hospital Centre Zagreb, Zagreb, Croatia; 4 Department of Medical Laboratory Diagnostics, University Hospital Centre Split, Split, Croatia; 5 Clinical Institute of Laboratory Diagnostics, Clinical Hospital Center Rijeka, Rijeka, Croatia; 6 Department of Medical Informatics, Rijeka University School of Medicine, Rijeka, Croatia

**Keywords:** informed consent, ethical approval, research, laboratory medicine, ethics committees

## Abstract

Informed consent is a process in which a human subject who is to participate in research needs to give his or her consent after being properly informed of the expected benefits as well as the potential harm of the research that will be performed. The function and purpose of the research ethics committee is to ensure that the research that will take place is in accordance with the relevant ethical standards. This means that the committee must assess the appropriateness of the design of the study reviewed. Research in the field of laboratory medicine has specific features, *i.e.* the use of samples that remain after routine analysis, data collection from databases containing patient information, data mining, collection of laboratory management data, method/instrument comparisons and validation, *etc.* As most of such research is either retrospective or not directly associated with patients, the question arises as to whether all types of research require informed consent and ethics committee approval. This article aims to clarify what is specific about obtaining informed consent and ethical approval in laboratory medicine, to provide general guidance on informed consent and ethical approval requirements based on the type of study, and what information should be included in applications for ethical approval and informed consent. This could also provide some guidance for future contributors to the *Biochemia Medica.*

## Introduction

Research is an activity which by using subjects has an intention that is cognitive by nature, and reflectively aims to provide logically formulated proof of a scientific hypothesis. The primary goal of researchers is the advancement of scientific knowledge. Beneficence and non-maleficence have different meanings in this case: the risks and benefits of research are weighed against their value for individuals, but also the greater good - scientific knowledge. This is why, in the first half of the 20^th^ century, the primacy of the supposed “greater good” over the destiny of an individual led to a series of infamous episodes involving experiments on human subjects (*1*, *2*). The consequences of these events were threefold: the introduction of the informed consent process for research participants, the introduction of research ethics committees, and the implementation of stringent rules, protocols and national/international legal standards regulating research in the field of biomedical sciences, especially research done on humans (*3*, *4*).

Informed consent is a process in which a human subject who is to participate in research needs to give his or her consent after being properly informed of the expected benefits as well as the potential harm of the research that will be performed (*5*). When giving consent, the subject needs to be legally capable of deciding. The subject should not be a minor or a person whose legal capacity is diminished. The person should not be forced in any way (physically, financially or emotionally/mentally) to participate in the research, and should decide freely. The process of obtaining informed consent is a process of information exchange. In it the subjects are informed clearly, and in a way that they understand: a) the nature of a research they would participate in, b) the potential benefits for him/her, a specific group of patients or society in general, and c) the potential harm for him/her (discomfort, painful procedures included in the research, *etc.*). After the subject has been provided with all the relevant information, he or she has to decide whether they want to participate in the research or not. If the answer is yes, they will confirm this by signing the consent form. It is important to stress that the subject may refuse to participate in the research or withdraw consent even after it has been given, and after the beginning of the research (*6*).

The research ethics committee (REC) or institutional review board (IRB) has the function of ensuring that the research design is in accordance with the relevant ethical standards (*7*). It also has to assess the appropriateness of the design of the study reviewed. The membership structure of a REC is interdisciplinary. The selection of members focuses on the competences of its members to assess the acceptability of research regarding its legal standards, professional practice and community acceptance (*8*). The constitution and functioning of the REC are in the majority of cases regulated by different types of legal provisions and implemented in various international documents (The Council for International Organizations of Medical Sciences (CIOMS) guidelines, the Helsinki Declaration, the Oviedo Convention of the Council of Europe, Regulation (EU) No 536/2014 of the European Parliament and of the Council) (*9*).

## What is the difference between informed consent for treatment and informed consent for research?

There is a difference between the informed consent process for treatment and the informed consent process in research. Informed consent obtained for the purpose of treatment is a result of the physician-patient therapeutic relationship. It is a discussion between equal partners (the physician and the patient) about the best possible treatment options for the patient. The patient is always a subject in this process, never an object (*10*).

The relationship between physician/researcher and patient/subject is guided by a different set of rules: the selection of suitable subjects and appropriate treatment of those subjects, careful application of research methodology, and acquisition of high-quality data, all with the purpose of proving a scientific hypothesis. While the purpose of ethics in the traditional physician-patient relationship is based on doing good, in this new physician/researcher-patient/subject relationship, high ethical standards are equated with the high quality of science. Informed consent in research implies that participants in the future research give the researcher permission to treat him/her for this specific research as an object of the research (*11*).

## What is specific about obtaining informed consent and ethics committee approval in laboratory medicine?

Informed consent in laboratory medicine can also, as in any other field of medicine, be obtained for two purposes: for a laboratory test that will lead to diagnosis and treatment if necessary, and for research. The consent of a patient in laboratory medicine, especially for simple diagnostic tests such as common blood work tests, is usually presumed. It is presumed when the patient gives his arm to the laboratory technician for blood to be drawn. However, for certain tests that may have significant consequences for the patients’ future, such as pregnancy or genetic testing, a well thought out process of informed consent is needed. A special approach to the informed consent process will also always be required for tests done on children or patients who are not able to give consent. Here their legal guardians have to be involved in the process. Children and patients not able to give consent must also be involved in the process, but only to the extent of their capacity to comprehend what is being done (*12*).

The informed consent process in research in laboratory medicine also has specific features since not all types of research in laboratory medicine require informed consent from the research participants or ethics committee approval (see the following Examples). Ethics committee approval is necessary when data are collected from databases which contain patient information. If patient data are anonymized, ethical approval is not required. More specifically, this applies to collecting laboratory management data which is not associated with patient information (*i.e.,* quality indicators, nonconformities in the pre- and postanalytical phase, turnaround-time, and test utilization). However, in the case of any genetic testing, even if the data are anonymous, ethics committee approval is required (*13*).

The obligation to obtain ethical approval can vary from institution to institution based on internal institutional review board guidelines. Researchers should always comply with such guidelines. However, general guidance on informed consent and ethical approval requirements, based on the study type, are listed in Table 1.

**Table 1:** Guidance on informed consent and ethical approval requirements based on the study type in the field of laboratory medicine

**Type of study**	**Study design**	**Informed consent**	**Ethical approval**
Research	The material from patients/healthy donors is collected for research purpose	Required	Required
Method/instrument validation	The use of residual material	Not required	Required
	The material from patients/healthy donors is collected for research purposes	Required	Required
Research showing standard clinical/laboratory practices or the advancement of the standard practices	If it does not include a new method or instrument	Not required (it is implied that informed consent was previously given for the scope of the treatment)	Not required (it is considered that this is not research but clinical/laboratory practice)
Incidence/epidemiological research	The use of residual material or retrospective data collection	Not required	Required
Laboratory information system (database) data extraction	Retrospective data collection	Not required	Required
Laboratory management	Studies that do not include human subjects, but collect data for measuring quality indicators (*i.e.* turnaround time, test utilization, non-conformities, *etc*.)	Not required	Not required
Survey	The participants are notified in the survey about the nature of the research and the future use of the data (publishing, *etc*.)	Not required (it is implied)	Not required
	Survey asking more intimate questions	Required	Not required
Case report	/	Required	Not required
Preanalytical case report	Patient specific information are not presented (patient is not identifiable)	Not required	Not required

***Example 1.*** To examine the diagnostic accuracy of protein X as a marker of cerebrospinal fluid leak, authors quantified protein X in blood and rhinorrhoea samples which were collected from patients. Quantification of protein X had been ordered by a medical doctor. The index test (protein X) is routinely performed in the laboratory.

Type of research (TR): Research (standard clinical/laboratory practice, advancement of standard practice).

Informed consent (IC): Not required.

Ethical approval (EA): Required.

Short elaboration: This study was conducted as part of standard clinical/laboratory practice and as such does not require informed consent. However, the results were retrospectively collected from the patient database, which required ethical approval.

***Example 2.*** The authors evaluated analytical performance of the assay X on instrument Y and measured the activity of enzyme Z in an adult population to establish reference intervals and evaluate correlation with age. Serum samples from the reference population were retrospectively selected from stored residual samples. These samples were obtained from healthy volunteers who had undergone a complete medical check-up and had provided informed consent for the collection, storage and reuse of their samples.

TR: Method validation.

IC: Required.

EA: Required.

Short elaboration: In this case, healthy volunteers provided their informed consent for the collection, storage and reuse of their samples. The volunteers were not aware in which exact studies their samples would be included. To ensure that these samples were going to be used in worthwhile research and handled properly, the authors needed to obtain ethical approval as well.

***Example 3.*** The authors conducted a study to validate a new type of tubes for plasma collection. The study included inpatients from the Intensive Care Unit (ICU) and randomly selected healthy volunteers. Blood samples from each individual were collected in three different tubes. The analytes’ concentrations in different tubes were compared with concentrations obtained in reference tubes.

TR: Method validation.

IC: Required.

EA: Required.

Short elaboration: Although verification of new test tubes is a standard laboratory practice, additional blood was collected from patients which required informed consent (*14*). Ethical approval was necessary because of the sensitive population included in the study (ICU patients). Similar to Example 2, method validation required study participants’ informed consent and ethical approval.

***Example 4.*** The study aimed to validate a new turbidimetric immunoassay on analyser X for measurement of protein Y in stool samples. Samples were obtained from a paediatric population of patients admitted to the hospital with specific gastrointestinal symptoms, and were retrospectively selected for method validation. A prerequisite for including individuals in the study was that a medical doctor had ordered a test for protein Y.

TR: Method validation.

IC: Not required.

EA: Required.

Short elaboration: In this case of method validation the test was ordered as part of standard clinical/laboratory practice. To ensure that these samples were to be used in worthwhile research and handled properly, the authors needed to obtain ethical approval.

***Example 5.*** The reference interval for protein X was changed in 2014, which made the interval narrower. The authors hypothesized that the change increased the prevalence of disease related to protein X and consequently caused greater economic costs. The authors extracted and anonymized patient data from a two-year period from the laboratory information system, obtaining 350,110 protein X concentration requests made by medical doctors.

TR: Data extraction from databases.

IC: Not required.

EA: Not required.

Short elaboration: Data were extracted from the database and anonymized, *i.e.,* data had already been obtained through a standard clinical/laboratory practice and were statistically analyzed in this study. Therefore, informed consent and ethical approval were not required.

***Example 6.*** The accuracy of mixing tests performed in samples with sporadically prolonged PT or APTT was retrospectively evaluated. Mixing studies are a standard laboratory practice for elucidating if the cause of a prolonged test is factor deficiency or the presence of an inhibitor.

TR: Data extraction from databases; standard laboratory/clinical practice.

IC: Not required.

EA: Required.

Short elaboration: Due to the retrospective nature of the study informed consent was not required. In contrast to Example 5, patient data were not anonymized and therefore ethical approval was required.

***Example 7:*** Unconscious patients treated in the emergency department and with a toxicology screen ordered by a medical doctor, were retrospectively inspected for substance abuse.

TR: Incidence research; data extraction from databases.

IC: Not required.

EA: Required.

Short elaboration: Due to the retrospective nature of the study informed consent was not required. Patient data were not anonymized and therefore ethical approval was required.

***Example 8:*** A retrospective study of every systematic examination blood test request was performed over a period of 6 months. The results were classified as unexpected if there was no previous pathological result present. Changes in patient management based on unexpected test results were retrieved from patients’ medical histories.

TR: Data extraction; standard clinical practice.

IC: Not required.

EA: Required.

Short elaboration: Due to the retrospective nature of the study informed consent was not required. Patient data were not anonymized and therefore ethical approval was required.

***Example 9:*** A test ordering form in the hospital information system was modified in order to rationalize test utilization and evaluate if this is potentially cost-saving. The number of patients tested and tests ordered during the 3 months following the introduced change were compared with the same period in the previous year.

TR: Data extraction; laboratory management.

IC: Not required.

EA: Not required.

Short elaboration: Laboratory management data were extracted from a database that does not contain patient-specific information. Therefore neither informed consent nor ethical approval are required.

## How to seek an approval from a research ethics committee

In order to successfully receive approval from a research ethics committee a research proposal outline should be submitted, as well as the informed consent form, if the research requires one.

## Research proposal outline

In order to obtain approval for a research proposal from the research ethics committee the research proposal must be submitted in full. This research proposal outline should consist of: the Hypothesis, Aims and purpose of the research, Materials, Subjects, Methodology, Research plan and Statistics. Explanation of the research proposal is important. The Hypothesis and Aims and purpose of the research serve as the basis for the discussion on the ethical acceptability of the research proposal involving humans, due to their role as the connecting points between the scientific and the ethical approaches to the work. Without a scientifically justified Hypothesis together with the Aims and purpose of the research, a favourable opinion on the ethical acceptability of the research proposal cannot be granted. Nonetheless, scientific justification does not guarantee ethical acceptability (*15*).

In addition to the Hypothesis and Aims and purpose of the research proposal, the other parts of the research proposal need to be explained in writing in a clear and detailed manner. The manner and the source of collection of results need to be clearly stated, whether it is biological material of human origin (*e.g.,* blood, serum, cerebrospinal fluid, urine, tissue gathered during an organ biopsy, *etc.*) or archived medical documentation, archived human biological material, specific diagnostic test results (*e.g.,* radiology), electronic databases, *etc.* (*16*)

The part regarding the subjects needs to be described very precisely. If the research is conducted (or was conducted) on one group of subjects, that group has to be defined according to the inclusion criteria. Also, the age range, gender and number of subjects per group need to be defined. If necessary, non-inclusion or exclusion criteria must be added to the inclusion criteria. The size of the sample is also important in relation to the hypothesis and the statistical relevance of the results gathered. The characteristics of each subject group (healthy or ill), such as age, gender, habits, as well as diagnosis and symptoms of patients, the duration of illness and the medication that they are currently receiving need to be defined (*17*).

When using patients’ archived medical data or archived human biological material as sources for data collection *(e.g*., frozen serum/plasma/cerebrospinal fluid samples or histological blocks with tumour tissue samples gathered after surgical procedures, which are stored in the archives of pathology laboratories), groups of data need to be clearly defined. Regardless of the source of future research material (subjects, medical documentation, archived human biological material), the proposal needs to state expressly how the integrity of the subjects will be protected and how the privacy of the gathered information will be ensured. It needs to be stated expressly how the gathered material will be used. If the participants are to be subjected to invasive diagnostic tests, these should be described in detail. The same rule applies to detailed lists of grading scales and criteria in trials on patients diagnosed with mental illnesses. If a survey is used as the primary research method, an example of the survey questionnaire needs to be provided (*18*, *19*).

Studies involving subjects (regardless of the source of collected data) may be divided into prospective and retrospective studies depending on the time criteria. In a retrospective study for example, the effects of drugs, the results of specific diagnostic tests or therapeutic treatments are analyzed over a specific period of time in the past. A prospective study is planned and executed according to a research plan set in advance, meaning that the parameters to be followed in one, two or more subject groups are precisely defined. If the research requires informed consent the process by which this will be obtained should be described and informed consent forms submitted (*15*).

## Informed consent forms

In prospective biomedical research involving humans, it is imperative to inform the subjects (healthy or ill) that they will be participating in research and obtain their consent. Informed consent needs to be translated into a language that the study participants can read and understand. It should be clearly written, easily understandable and not excessively lengthy. Vocabulary that most participants would not understand should be avoided (not all subjects are medical workers or have high levels of education, *etc*.). Excessive detail should also be avoided (*20*, *21*).

Informed consent commonly includes a short invitation in which the purpose of the research is described. Then the hypothesis and aims of the research protocol are explained in a concise and clear manner. There should be a part regarding the subjects’ participation in the research. This part should clearly describe the role of the subjects in the research, the tests that they will be subjected to, and the quantities of samples to be extracted for the research. If the diagnostic procedure includes organ biopsy, the subjects should be asked whether they consent to having part of their sample used not only for diagnostic purposes, but also stored and used for future scientific research. The same consent should also be obtained when collecting organ samples in surgical procedures or collecting any other kind of biological samples. These types of consent are particularly important for research which is not yet planned; stored materials might become interesting in the future due to later scientific discoveries and methodological approaches (archived biological materials and retrospective research) (*19*). Furthermore, the subject has the right to know the expected advantages and benefits of participation in the research, as well as the potential risks and adverse events. If future generations will benefit from the research results, but there are no direct benefits for the subject, that should be made clear as well. In some research proposals, the subjects are to be exposed to invasive diagnostic and/or therapeutic procedures. Each researcher must list the risks and benefits of each of those procedures and enclose statistical data concerning the risk to benefit ratio in the Informed consent form (*22*).

The subject may refuse to participate in a trial without consequences. If a trial involves children under the age of 18, written informed consent is required from their parent/guardian. For children above 10 years of age a signed assent form is also required (*23*).

If genetic material is used in research, the Informed consent form needs to include the following information:

what exactly will be done with the samples
how long the samples will be stored (months, years) and when they will be destroyed,
the research in which the samples will be used,
the Informed consent form needs to state explicitly that the samples will only be used in that research
if the samples will be used in other research, informed consent needs to be obtained from the donor of the sample for each new use
for each new genetic research using a previously collected sample, the opinion of the Working Party on Ethical Acceptability needs to be acquired
in the Informed consent form, consent for RNA analysis and consent for DNA analysis need to be separated clearly in order to provide the subjects with the option to choose the type of analysis they consent to
since research involving genetic material may result in possible incidental findings *(e.g.* finding a gene for a hereditary disease), the Informed consent form needs to state that the owners of the samples are free to choose to have the incidental findings reported to them or not. Therefore, a statement with a positive or negative response that the owner of the sample needs to sign needs to be included at the end of the Informed consent form.
if the material is to be processed in institutions outside the country where the material is collected, the Informed consent form needs to state whether the rest of the genetic material will be stored abroad and whom the material will belong to upon completion of the research (*24*).

The Informed consent form needs to define how the confidentiality of the subjects’ records (personal and medical) will be protected, and to what extent the information gathered in the research will be used. Furthermore, it is necessary to define who evaluated and approved the research and whom to contact for additional information. Finally, the Informed consent form should end with a statement by the subjects confirming that they fully understand their involvement in the research. The consent is to be signed by the researcher and the subject (or parent/guardian). Some also recommend that an additional witness should be present and also sign the Informed consent form, but not all authors agree that this is necessary (*25*, *26*).

## Conclusion

This article outlines what is specific about obtaining informed consent and ethical approval in laboratory medicine. It provides general guidance on informed consent and ethical approval requirements, based on the type of study, and what information should be included in the application for ethical approval and informed consent. This contribution could also provide guidance for future contributors to the *Biochemia Medica* since it is committed to ensure and promote the best practice guidelines given by Committee on Publication Ethics (COPE) (available at: http://publicationethics.org/files/Code%20of%20Conduct_2.pdf). According to these guidelines, journals should encourage ethical research and protect individual data obtained in the course of research (*27*). Therefore, all manuscripts submitted to the *Biochemia Medica* are considered closely regarding the question of ethical research.

Upon submission, the authors must complete a questionnaire where, among other questions, they state:

if the reported research was approved by an institutional/national ethics committee; if it was, which one; if not, an explanation is needed
if appropriate informed consent was obtained from each research participant; if not, an explanation is needed
if the authors obtained the patients’ written permission or the approval of next-of-kin to report a submitted Case report or another type of article where the patient is identifiable.

The authors are encouraged to consult Table 1 for guidance.
